# Effect of Vertical Annealing on the Nitrogen Dioxide Response of Organic Thin Film Transistors

**DOI:** 10.3390/nano8040203

**Published:** 2018-03-29

**Authors:** Sihui Hou, Xinming Zhuang, Zuchong Yang, Junsheng Yu

**Affiliations:** State Key Laboratory of Electronic Thin Films and Integrated Devices, School of Optoelectronic Science and Engineering, University of Electronic Science and Technology of China (UESTC), Chengdu 610054, China; 201621050313@std.uestc.edu.cn (S.H.); xinmingzhuang@std.uestc.edu.cn (X.Z.); 201621050314@std.uestc.edu.cn (Z.Y.)

**Keywords:** organic thin-film transistor (OTFT), vertical annealing process, grain boundary, nitrogen dioxide (NO_2_), gas sensor

## Abstract

Nitrogen dioxide (NO_2_) sensors based on organic thin-film transistors (OTFTs) were fabricated by conventional annealing (horizontal) and vertical annealing processes of organic semiconductor (OSC) films. The NO_2_ responsivity of OTFTs to 15 ppm of NO_2_ is 1408% under conditions of vertical annealing and only 72% when conventional annealing is applied. Moreover, gas sensors obtained by vertical annealing achieve a high sensing performance of 589% already at 1 ppm of NO_2_, while showing a preferential response to NO_2_ compared with SO_2_, NH_3_, CO, and H_2_S. To analyze the mechanism of performance improvement of OTFT gas sensors, the morphologies of 6,13-bis(triisopropylsilylethynyl)-pentacene (TIPS-pentacene) films were characterized by atomic force microscopy (AFM) in tapping mode. The results show that, in well-aligned TIPS-pentacene films, a large number of effective grain boundaries inside the conducting channel contribute to the enhancement of NO_2_ gas sensing performance.

## 1. Introduction

In the process of human production and life, a large number of toxic and harmful gases, such as nitrogen dioxide (NO_2_), sulfur dioxide (SO_2_), and ammonia (NH_3_), are unavoidably emitted into the atmosphere. Harmful gases not only damage the ecological environment, but also pose a serious threat to human health [1,2,3]. Therefore, it is extremely important to realize an effective monitoring of the atmospheric environment. Compared with other traditional gas sensors, as shown in Table 1, organic thin-film transistor (OTFT) gas sensors have become a research hotspot because of their low-cost, room-temperature operation, high sensitivity, and simple fabrication [4,5,6,7,8,9,10,11]. Besides, multi-parameter detection, such as mobility (*µ*), threshold voltage (*V_T_*), and electrical conductivity (*σ*), favors to the development of fully automatic digital intelligent gas sensors that can detect various gases at the same time [12,13]. Film morphology is an important factor affecting those parameters of OTFTs. By specific process engineering, well-aligned organic semiconductor (OSC) films can be obtained, in which the backbone of molecules interconnects in a certain orientation by weak non-covalent bonding interactions [14]. This method of morphology modification not only effectively controls the performance of OTFTs, but also has great potential to improve the sensitivity of gas sensors. However, it’s still a challenge to get well-aligned OSC films.

In recent years, many studies have focused on the formation of highly ordered and well-aligned active layers by different process engineering, and there are two main schemes that have been developed for solution processed of OTFTs [15]. One is solution permeation by pre-designed templates in the coating process, such as zone casting, rotation coating, dip coating, blade coating, brush coating, and meniscus-guided coating, and the related reported processes and references are shown in Table 2 [16,17,18,19,20,21]. The other is pre-patterning separated wetting and dewetting regions prior to the annealing process. Bharti et al. obtained high performance OTFTs with highly aligned and crystalline OSC films by a directional solvent vapor annealing method, making the mobility increase from 0.29 cm^2^/Vs to 0.86 cm^2^/Vs. [22]. Keum et al. achieved well-aligned 6,13-bis(triisopropylsilylethynyl)-pentacene (TIPS-pentacene) films by applying an anisotropic temperature-gradient during the process of solvent drying, making the mobility about four-times higher [23]. Li et al. took advantage of a tiny slope and rapid annealing to fabricate highly-ordered single crystal arrays in desired orientation [24]. In addition to effectively improving the performance of OTFTs, well-aligned OSC films also show great potential for improving the gas sensing performance.

So far, many research groups have focused on the effect of well-aligned OSC films on the gas sensing performance of OTFTs by applying specific process engineering to the coating process. Li et al. fabricated an ammonia gas sensor with an ultrathin well-aligned OSC film, which provides direct and efficient pathways for achieving improved sensing performance [25]. Wang et al. obtained an active layer with an orientated micro-stripes structure by combining a fast dip-coating method and simple surface engineering, getting a high sensitivity of about 160 under 50 ppm NH_3_ [26]. Although many studies have focused on fabricating well-aligned OSC films by special coating processes to improve the gas sensing performance of OTFTs, the gas sensing performance of OSC film, well-aligned during annealing, has not yet received much attention. Furthermore, there are many researches about the enhancement of NO_2_ sensing performance. Shi et al. obtained a response of 53.6% to 50 ppm NO_2_ by introducing DNA as an interlayer [27]; and Wang et al. utilized para-sexiphenyl to modify the interface of dielectric/OSC films and shown a response of 6300% to 5 ppm NO_2_ [28]. Huang et al. also reported NO_2_ sensors with the dielectric surface UV–ozone treatment which exhibited high sensitivity of 16,000% to 30 ppm NO_2_ [1]. In these studies, additional functional layers or processes are used to improve the gas sensing performance, which inevitably leads to the enhancement of process complexity and energy consumption. Thus, how to obtain high performance NO_2_ sensors using a simple process is still a challenge.

In this work, we fabricated OTFT based NO_2_ gas sensors by both vertical annealing and conventional annealing processes. As a result, different electrical performances were obtained by these two annealing methods, and the NO_2_ sensing performances of OTFTs were analyzed in detail. The gas sensors formed by conventional and vertical annealing processes show significant differences in their gas sensing performance. OTFTs formed by vertical annealing show a response of 1408% at 15 ppm NO_2_ as compared to 72% of conventional annealed devices, thus achieving a 20-fold improvement. A large number of effective grain boundaries in the conducting channel are mainly responsible for the enhanced sensing performance of the device. This simple vertical annealing process promises to be an effective method for the fabrication of high performance OTFT based NO_2_ sensors. Additionally, it offers great potential for the industrial manufacture of low-cost, fast, and portable electronic noses.

## 2. Experimental

### 2.1. Device Preparation

**Figure 1:**
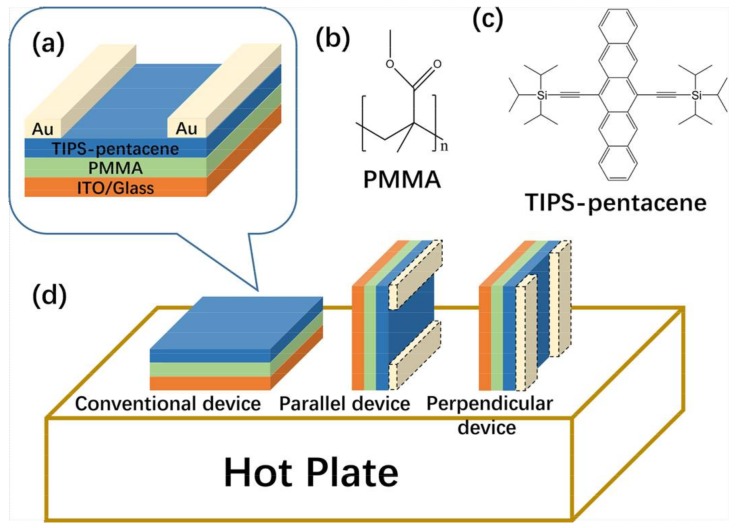
Device architecture of the OTFTs used in this study (**a**). Molecular structures of PMMA (**b**) and TIPS-pentacene (**c**). Schematic of OTFT subjected to different annealing processes (**d**).

The OTFFs were fabricated on indium tin oxide (ITO) glass substrates. The architecture of the OTFTs is shown in Figure 1, along with the chemical structures of TIPS-pentacene OSC and PMMA dielectric materials. Before the dielectric layers were spin-coated, the substrates were ultrasonically cleaned first in acetone, and then in deionized water and isopropyl alcohol for 15 min each, followed by an UV-ozone treatment for 10 min. PMMA (average Mw~120,000, 10 wt. %) was dissolved in anisole. The dielectric polymer was spin-coated on the ITO substrates at room temperature and dried in an oven at 135 °C for 1 h. The OSC material Tips-pentacene (Mw~280,000, 5 mg mL^−1^ in 1,2-dichlorobenzene) was spin-coated on the PMMA dielectric layer at 3000 rpm for 60 s (about 25 nm). The device was then placed on a hot plate at 125 °C for 15 min to completely remove any residual solvents. The devices were placed horizontally and vertically onto the hot plate surface, and the devices that were vertically placed were divided into two groups with the electrodes, either parallel or perpendicular to the hot plate surface. In the following we will call these simply parallel and perpendicular devices, respectively. The specific annealing methods are shown in Figure 1. Subsequently, the finger source/drain electrodes (30 nm) were deposited under 3 × 10^3^ Pa through a shadow mask. The electrical characteristics of the OFETs were carried out with a Keithley 4200 sourcemeter (Keithley, Cleveland, OH, USA) in nitrogen at room temperature. Charge carrier mobility (*µ*) and threshold voltage (*V_T_*) were extracted in the saturation regime from the highest slope of |*I_DS_*|^1/2^ vs. *V_GS_* plots using the following Equation [29]:

*I_DS_* = (*W*/2*L*)*µC*(*V_GS_* − *V_T_*)^2^, (1)

where *L* (100 µm) is the channel length, *W* (1 cm) is the channel width, *C* is the capacitance (per unit area) of the dielectric, *V_GS_* is the gate voltage, and *I_DS_* is the drain-source current.

### 2.2. Film Characterization and Sensor Test

The morphologies of the TIPS-pentacene films were characterized by atomic force microscopy (AFM; Agilent, Santa Clara, CA, USA) in tapping mode. The Fourier transform infrared (FTIR) measurements were conducted with a FTIR spectrometer (Thermo Scientific, Nicole-10, Waltham, MA, USA). Before testing, the OTFT-sensors were deposited in a nitrogen environment. For sensor tests, the devices were stored in an airtight test chamber (approximately 16 mL). Dry air, 50 ppm standard NO_2_ and H_2_S gases and 100 ppm standard NH_3_, SO_2_ and CO gases were purchased from Sichuan Tianyi Science & Technology Co. (Chengdu, China), and a mixture with the appropriate concentrations was introduced into the test chamber by mass flow controllers (S48 300/HMT, Beijing BORIBA METRON Instruments Co., Beijing, China). The flow rate during the test was fixed at 100 sccm (standard cm^3^ per min). NO_2_ gas response characteristics of OTFT-sensors were measured with *I_DS_* varying as a function of time, with an operating temperature of 300 K, the response time of 800 s and the recovery time of 700 s. Also, the transfer curves in various concentrations of NO_2_ were characterized.

## 3. Results and Discussion

**Figure 2:**
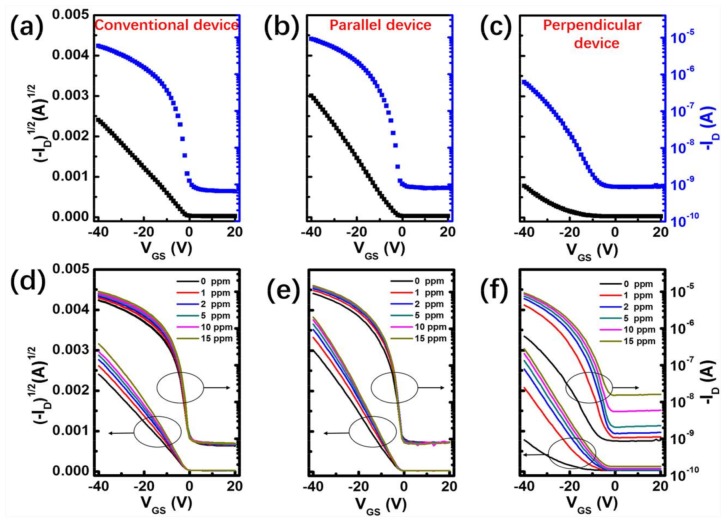
(**a**–**c**) Transfer characteristics curves of OTFTs obtained by two different annealing methods. Transfer curves (**d**–**f**) of OTFT sensors obtained by different annealing methods under a specific concentration of NO_2_ (*V_DS_* = −40 V).

The electrical performances of OTFTs obtained by the two different annealing methods are firstly investigated. Transfer characteristics of the devices are presented in Figure 2, with a source-drain voltage (*V_DS_*) of −40 V. It can be seen that different annealing processes have obviously changed the electrical performances of the OTFTs, including mobility (*µ*), on-off ratio (*I_on_*/*I_off_*), threshold voltage (*V_T_*), and subthreshold slope (*SS*), and the detailed electrical parameters of three types of OTFTs are listed in Table 3.

As shown in Table 3, we can see that the parallel device reveals an enhanced *µ* of 0.0275 cm^2^/Vs, which is higher than the mobilities of the conventional (0.0186 cm^2^/Vs) and the perpendicular (0.0092 cm^2^/Vs) devices. The corresponding saturation current (*I_on_*) are 9.03 µA, 5.79 µA, and 0.61 µA, respectively. Moreover, the parallel and conventional devices have the same *I_on_*/*I_off_* of about 10^4^, while the *I_on_*/*I_off_* of the perpendicular device is only about 10^3^. In general, the parallel device shows the best electrical performance, and there is an obvious difference in electrical performance between parallel and perpendicular devices obtained by the same annealing process.

**Figure 3:**
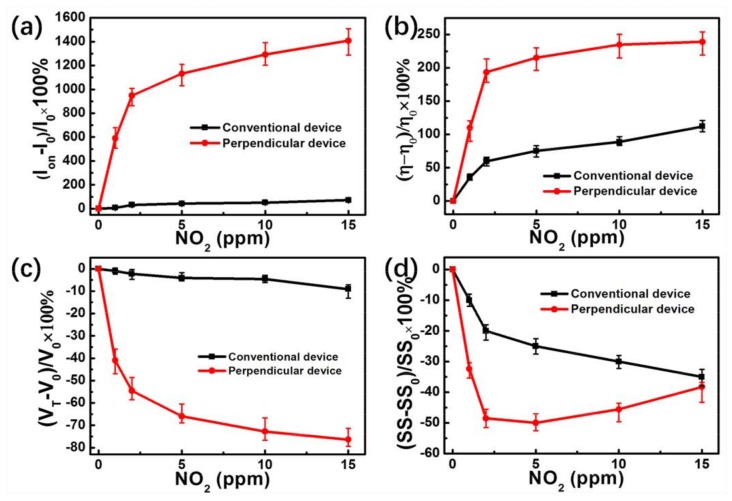
Variation of *I_on_* (**a**), *µ* (**b**), *V_T_* (**c**), and *SS* (**d**) of OTFTs at different NO_2_ concentrations.

Based on the above improved performance of OTFTs, the gas sensing performance of the devices with conventional annealing and vertical annealing are also characterized by measuring transistor characteristics under programmed NO_2_ exposure ranging from 1 to 15 ppm. The transfer characteristics of the OTFTs exposed to various concentrations of NO_2_ are shown in Figure 2. For the transfer curve measurements, all devices were exposed to a specific concentration of NO_2_ for 2 min before measurement. After introducing strongly oxidizing NO_2_, some electrons in OSC film will become trapped. Thus, the number of free holes increase, resulting in a reduction of the potential barriers, and an enhancement in the source-drain saturation current [27]. Figure 2a,b show that parallel devices exhibit smaller responses of *I*_on_ increasing from 9 µA to 14.7 µA (63%) when exposed to 15 ppm NO_2_, in contrast to conventional devices which increased from 5.8 µA to 10 µA (72%). However, as shown in Figure 3c, the *I_on_* of the perpendicular device increases significantly from 0.6 µA (0 ppm NO_2_) to 9.2 µA (15 ppm NO_2_), yielding a high responsivity of up to 1408%. In order to further understand the effect of different annealing processes on the gas sensing performances of OTFTs, we analyze the sensing performances of conventional and perpendicular devices in detail, including *I_on_*, *µ*, *SS*, and *V_T_*.

To assess the response *R* of the OTFT parameters *I_on_*, *µ*, *SS*, and *V_T_* to different NO_2_ concentrations we define *R* = [(*R*_NO2_ − *R*_AIR_)/*R*_AIR_] × 100%. From the data presented in Figure 3a, it can be seen that *I_on_* of the perpendicular device increased by 1408%, when exposed to 15 ppm NO_2_. In comparison, the conventional device only increased by 72% under the same concentration of NO_2_. Accordingly, the perpendicular device shows a greater *µ* variance rate of 239% in 15 ppm NO_2_ as compared to 112% of the conventional device. After NO_2_ exposure, the *V_T_* of these two kinds of devices experience only a slight positive shift, while the perpendicular one experiences a much more significant change as shown in Figure 3c. As *V_T_* shifts are usually caused by charge trapping at the dielectric/semiconductor interface, more NO_2_ molecules will trap at the interface and cause more immobilized negative charges to become located there. These localized charges, in turn, will induce more free holes and reduce the negative gate voltage required for the transistor turn-on [30]. Furthermore, as *SS* is proportional to the trap density at the dielectric/semiconductor interface, the trap density (*N*) can be extracted by Equation (2) [31,32]:

*SS* = *kT*/*q*ln10(1 + *qN*/*C*), (2)

where *q* is the electronic charge, *k* is Boltzmann’s constant, *T* is the absolute temperature, and *C* is the areal capacitance of the dielectric structure. As shown in Figure 3d, the relative change in *SS* of the two devices decrease in a continuous manner as the NO_2_ concentration increases. The perpendicular device, in contrast, shows early saturation at fairly small values. We can see that the morphology of the OSC film is effectively affected by the different annealing processes, and that the density of the trap is a key factor determining the NO_2_ sensing performance.

**Figure 4:**
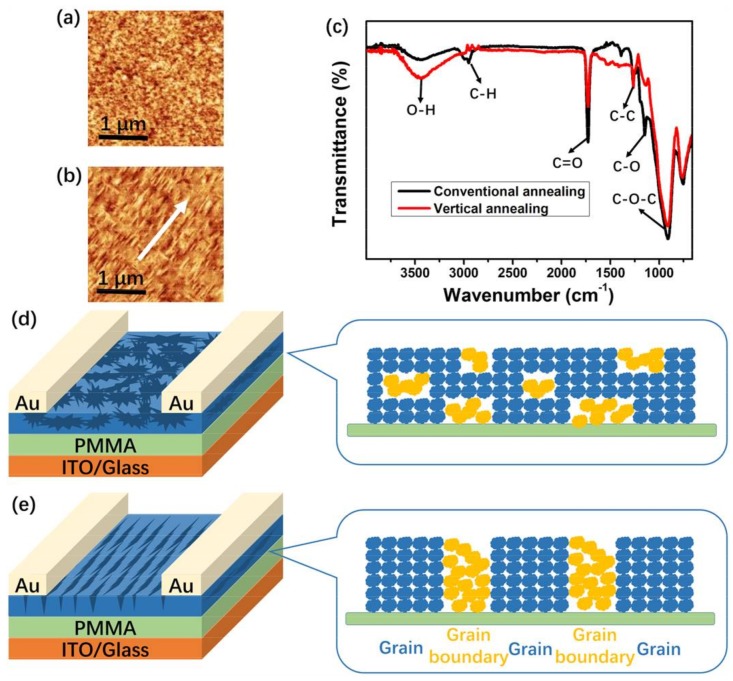
AFM topography images of TIPS-pentacene films of conventional device (**a**) and the perpendicular device (**b**). FTIR spectra of TIPS-pentacene films with the PMMA dielectric formed by conventional and vertical annealing methods (**c**). Schematic diagrams of crystallization orientation of parallel device (**d**) and the perpendicular device (**e**).

To elucidate the origin of the different gas sensing performances, AFM was utilized to analyze the morphology of the TIPS-pentacene films grown by conventional and vertical annealing processes. As shown in Figure 4a,b, well-ordered TIPS-pentacene films are formed on PMMA dielectrics by vertical annealing as compared to the irregular grains formed by conventional annealing. Furthermore, the similar FTIR spectra in Figure 4c indicate that there is almost no change in the chemical characteristics of the TIPS-pentacene films formed by two different annealing methods. Thus, well-aligned TIPS-pentacene films formed by vertical annealing are the major factor on the enhancement of NO_2_ sensing performance. Due to the well-aligned TIPS-pentacene films, the conductivity of active layers is highly anisotropic [33]. When the grain orientation of the OSC films is perpendicular to the conducting channel, charge carriers pass through a larger number of grain boundaries on their journey from source to drain electrodes than in a conventional device. In the band bending model, grain boundaries are represented by depletion regions, and the holes transfer via hopping from one grain to another [24,34]. This is in good agreement with the above phenomenon that perpendicular devices show a lower *µ* compared to conventional ones. Moreover, as illustrated in Figure 4d,e, in conventional device consisting of disordered TIPS-pentacene grains, charge carriers in the first monolayer are able to move through grains in the second layer, which short-circuit the grain boundaries in the first layer. In this way, grain boundaries become less important to charge transport, and the signals produced by the interaction of NO_2_ with these grain boundaries will not be shown in source-drain saturation current. In contrast, when moving in well-aligned TIPS-pentacene grains in vertical devices, charge carriers are inevitably forced into grain boundaries which cannot be bypassed through upper grains, thus resulting in larger grain boundary effects and a larger NO_2_ responsivity [35,36].

**Figure 5:**
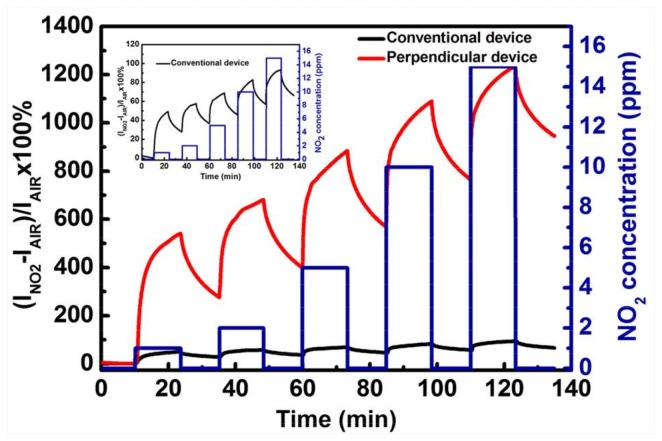
Response curves of conventional and perpendicular devices to NO_2_ pulses.

Furthermore, real-time responsivities were tested for three devices biased at *V_DS_* = *V_GS_* = −40 V, responding to the dynamic switching to different concentrations of NO_2_ (1, 2, 5, 10, and 15 ppm) at room temperature. Prior to each device test, the *I_DS_* of all devices was allowed to reach a steady state after 30 min in dry air. From the test results shown in Figure 5, it is obvious that the sensing performance of the perpendicular device is superior to an OTFT formed by conventional annealing. When exposed to 15 ppm NO_2_ for 10 min, the *I_DS_* charges in the conventional and perpendicular devices are 93% and 1235%, respectively. Compared to the conventional device, the perpendicular device shows a one-order-of-magnitude improvement in NO_2_ sensing performance. These results are in agreement with the previous results. Thus, the increased number of effective grain boundaries can effectively improve the NO_2_ sensing performance of OTFTs. The responses of conventional and parallel devices to NO_2_ pulses were also studied, and the obtained curves are shown in Figure S1 (Supplementary Materials). In addition, the perpendicular device shows a high responsivity of 539% already at a concentration of 1 ppm, which indicates that the vertical device offers great potential for becoming low-concentration detectors for NO_2_. Subsequently, the responses of conventional and perpendicular devices to lower concentration of NO_2_ (200, 400, 800 ppb) were tested (Figure S2, Supplementary Materials). The results exhibit that the perpendicular device also shows a greater response than the conventional device.

**Figure 6:**
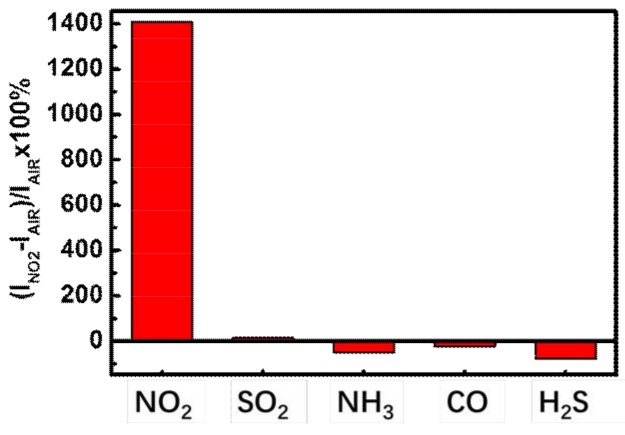
Drain current changes for perpendicular device exposed to different gases.

Selectivity is a crucial parameter for practical sensing applications, which usually relies on the specific interaction between the organic semiconductors and the analytes. To probe the selectivity of the device, we measured the responses of the perpendicular device to several other common gases, including SO_2_ as an oxidizing gas and NH_3_, CO, and H_2_S as reducing gases. All these gases are separately mixed with dry air to form a fixed concentration of 15 ppm. As shown in Figure 6, the perpendicular device presents a specifically preferential response to NO_2_, while no obvious responses are observed with 15 ppm SO_2_. The possible reason might be that NO_2_ is more strongly oxidizing on TIPS-pentacene than SO_2_ [37]. For the reducing gases of NH_3_, CO, and H_2_S, the saturation currents decrease with a decrement of −52%, −24%, and −77%, respectively. As a consequence, the other gases may be have weaker chemical interaction with TIPS-pentacene, resulting in a high responsivity to NO_2_.

## 4. Conclusions

In summary, a simple vertical annealing process was proposed for the fabrication of high-performance OTFT based NO_2_ sensors. The NO_2_ responsivity of OTFT based sensors improved one order of magnitude from 72% to 1408%, relative to conventionally prepared devices. The sensing performance enhancement is due to the superior alignment of TIPS-pentacene film caused by the vertical annealing process. In this way, a large number of effective grain boundaries in the conducting channel could be obtained, resulting in an improvement in the gas sensing performance. Moreover, gas sensors formed by vertical annealing achieved a high sensing performance of 589% already at 1 ppm NO_2_, showing at the same time a preferential response to NO_2_. The method of vertical annealing, demonstrated in this work, not only opens an effective way for the improvement of OTFT gas sensing performance, but also offers great potential for low-cost, fast, and portable electronic noses.

## Figures and Tables

**Table 1:** Properties of some common used gas sensors.

Test Method	Sensitivity	Selectivity	Response Speed	Cost	Detection Range	Ref.
OTFTs	excellent	good	great	low	ppb	[4,5,6,7]
Infrared Absorption	great	good	good	common	wide	[8]
Photochemistry	great	great	great	common	ppm	[9]
Chromatography	excellent	excellent	excellent	expensive	ppm	[10]
Resistive	excellent	good	good	low	ppm	[11]

**Table 2:** Recently-reported processes to obtain ordered active layers.

Author	Technique	Semiconductor	Solvent	Mobility (cm^2^ V^−1^ s^−1^)	Ref.
Su	Zone casting	TIPS-pentacene	Chloroform	0.67	[16]
Van Tho	Rotation coating	DPPT-TT	DCB	1.95	[17]
Nam	Dip coating	TIPS-pentacene	DCM	0.24	[18]
Niazi	Blade coating	diF-TES-ADT	toluene	6.70	[19]
Lin	Brush coating	DPPDTT	DCB	11.20	[20]
Janneck	Meniscus-guided coating	C_8_-BTBT	heptane	7.00	[21]

**Table 3:** Field-effect mobility (*µ*), current on-off ratio (*I_on_*/*I_off_*), threshold voltage (*V_T_*), and subthreshold slope (*SS*) of different OTFTs.

Device	*µ* (cm^2^/Vs)	*I_on_*/*I_off_*	*V_T_* (V)	*SS* (V/dec)
Conventional device	0.0186	~10^4^	−2.4	2.0
Parallel device	0.0275	~10^4^	−2.0	1.8
Perpendicular device	0.0092	~10^3^	−20.0	6.8

## Supplementary Materials

The following are available online at http://www.mdpi.com/2079-4991/8/4/203/s1, Figure S1: Response curves of the conventional and parallel device to NO_2_ pulses, Figure S2: Response curves of conventional and perpendicular devices to low concentration NO_2_ pulses.
