# Supplementary material for: Effect of Vertical Annealing on the Nitrogen Dioxide Response of Organic Thin Film Transistors
Source: Nanomaterials (Basel). 2018 Mar 29;8(4):203. doi: 10.3390/nano8040203 (PMC5923533; doi:10.3390/nano8040203)
Supplement: Supplementary file 1 [file nanomaterials-08-00203-s001.pdf]

Effect of vertical annealing on the nitrogen dioxide response of organic thin film transistors

Sihui Hou, Xinming Zhuang, Zuchong Yang, Junsheng Yu*

State Key Laboratory of Electronic Thin Films and Integrated Devices, School of Optoelectronic Science and Engineering, University of Electronic Science and Technology of China (UESTC), Chengdu 610054, PR China

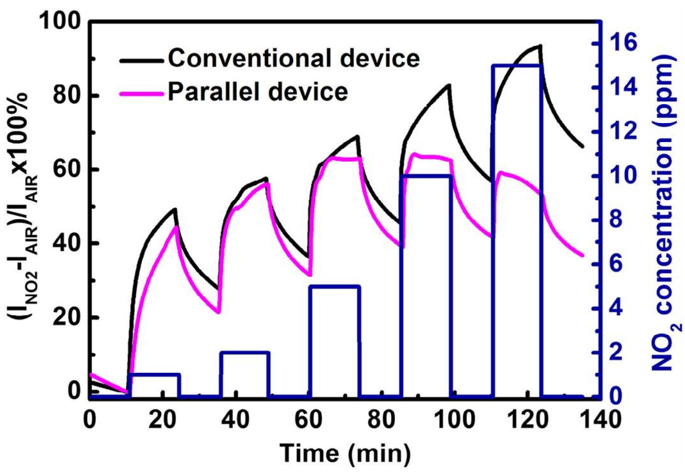

Figure S1. Response curves of the conventional and parallel devices to NO_2 pulses.

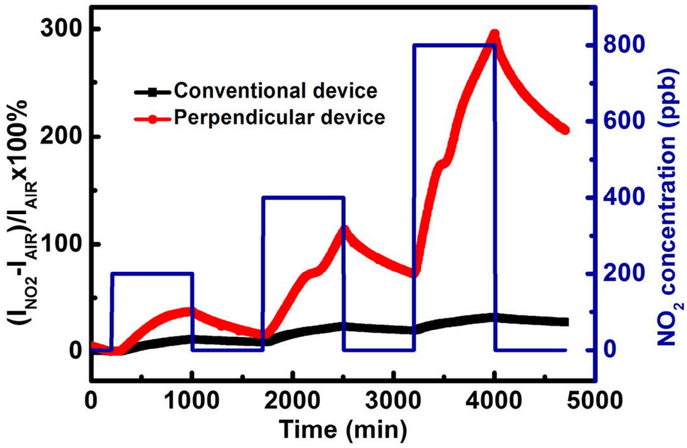

Figure S2. Response curves of the conventional and perpendicular devices to low concentration NO_2 pulses.

* Corresponding author.
E-mail addresses: jsyu@uestc.edu.cn (J.Yu).
